# Mechanical Stress Regulates Osteogenesis and Adipogenesis of Rat Mesenchymal Stem Cells through PI3K/Akt/GSK-3*β*/*β*-Catenin Signaling Pathway

**DOI:** 10.1155/2017/6027402

**Published:** 2017-02-14

**Authors:** Fanglong Song, Dawei Jiang, Tianchen Wang, Yi Wang, Yi Lou, Yinquan Zhang, Hui Ma, Yifan Kang

**Affiliations:** Department of Orthopedics, Third Affiliated Hospital, PLA Second Military Medical University, Shanghai 200433, China

## Abstract

Osteogenesis and adipogenesis of bone marrow mesenchymal stem cells (BMSCs) are regarded as being of great importance in the regulation of bone remodeling. In this study, rat BMSCs were exposed to different levels of cyclic mechanical stress generated by liquid drops and cultured in general medium or adipogenic medium. Markers of osteogenic (Runx2 and Collagen I) and adipogenic (C/EBP*α*, PPAR*γ*, and lipid droplets) differentiation were detected using Western blot and histological staining. The protein levels of members of the phosphatidylinositol 3-kinase (PI3K)/Akt/glycogen synthase kinase 3*β* (GSK-3*β*)/*β*-catenin signaling pathway were also examined. Results showed that small-magnitude stress significantly upregulated Runx2 and Collagen I and downregulated PPAR*γ* and C/EBP*α* expression in BMSCs cultured in adipogenic medium, while large-magnitude stress reversed the effect when compared with unloading groups. The PI3K/Akt signaling pathway could be strongly activated by mechanical stimulation; however, large-magnitude stress led to decreased activation of the signaling pathway when compared with small-magnitude stress. Activation of *β*-catenin with LiCl led to increased expression of Runx2 and Collagen I and reduction of C/EBP*α* and PPAR*γ* expression in BMSCs. Inhibition of PI3K/Akt signaling partially blocked the expression of *β*-catenin. Taken together, our results indicate that mechanical stress-regulated osteogenesis and adipogenesis of rat BMSCs are mediated, at least in part, by the PI3K/Akt/GSK-3*β*/*β*-catenin signaling pathway.

## 1. Introduction

Mesenchymal stem cells (MSCs) have multipotent differentiation capabilities and have been confirmed to differentiate into various lineages, including osteoblasts, adipocytes, chondrocytes, myocytes, and fibroblasts [1, 2]. Thus they hold great potential for regenerative medicine and tissue-engineering applications. It has been demonstrated that differentiation of MSCs into these various lineages is controlled by their particular biochemical microenvironment [2]. Additionally, mechanical stress has also been demonstrated to aid MSCs differentiation into various mature cells [3, 4]. Tension [5] and fluid shear stress [6, 7] have both been reported to promote osteogenic lineage commitment, while compression [8, 9] and hydrostatic pressure [10] increase both chondrogenic and osteogenic differentiation.

In bones, osteoblasts and adipocytes are derived from a common progenitor cell, the bone marrow MSCs (BMSCs) [11]. In particular, osteogenic differentiation of BMSCs is essential for the maintenance of bone quality and quantity. However, reduced osteogenic potential and relatively increased adipogenic differentiation are associated with various pathological conditions such as osteoporosis, resulting in reduced bone mass, increased bone fragility, and increased susceptibility to fracture [12]. Therefore, the commitment of BMSCs to the adipocyte and osteoblast lineages appears to be of great importance in the regulation of bone remodeling.

It has been widely accepted that physiological loading is beneficial in maintaining skeletal integrity and increasing bone strength [13], while osteoporosis or bone resorption is often observed under unloading conditions such as in bedridden patients, in conditions of microgravity, following fracture of a limb and in other conditions of skeletal unloading [14, 15]. Concentration of excessive mechanical stress often induces fatigue fractures and periprosthetic fractures. Physiological mechanical stress enhances the proliferation, differentiation, and extracellular matrix formation of osteoblasts [16, 17], while pathological mechanical loading has been reported to exert an apoptotic effect on osteoblasts [18–20], leading to different outcomes of bone metabolism and bone strength.

The commitment of MSCs to different lineages in response to mechanical stress results from different types, magnitudes, and/or frequencies of forces [21]. Intensive studies using different types of stress such as tensile stretch, fluid flow, compression, and cyclic hydrostatic pressure have been reported to induce bone formation and limit adipogenesis of MSCs, which occur as a concerted response [22]. However, much more remains to be discovered about the effect of different levels of mechanical stimulation on the adipogenic and osteogenic differentiation of MSCs.

Over decades of study, it has become more and more clear that the adipogenic differentiation and osteogenic differentiation of MSCs are competing and reciprocal [23]. Our previous research has demonstrated that the apoptotic or antiapoptotic effect of mechanical stress on the osteoblast depends on the levels of the stress loading on it [20]. Just as different apoptotic effects on osteoblasts are observed in response to different levels of mechanical stress, we hypothesized that osteogenic and adipogenic differentiation of MSCs may reverse with changes in the levels of mechanical loading. The purpose of the present study was to investigate the effects of different magnitudes of cyclic mechanical stress on the osteogenic and adipogenic differentiation of BMSCs and to clarify the underlying mechanism. Markers of osteogenic and adipogenic differentiation were observed in MSCs stimulated by cyclic compression stress generated by liquid drops, cultured in both general and adipogenic medium in vitro. Furthermore, changes in the phosphatidylinositol 3-kinase (PI3K)/Akt/glycogen synthase kinase 3*β* (GSK-3*β*)/*β*-catenin signaling pathway were investigated to illustrate the mechanism. Our present study thus provides a preliminary insight into the mechanical stress-induced changes of osteogenic and adipogenic differentiation in BMSCs and illustrates the role played by the PI3K/Akt/GSK-3*β*/*β*-catenin signaling pathway in the process.

## 2. Materials and Methods

### 2.1. Antibodies and Reagents

Antibodies against Runx2, C/EBP*α*, phosphor-Akt (p-Akt), Akt, GSK-3*β*, *β*-actin, and the appropriate anti-rabbit horseradish peroxidase- (HRP-) conjugated secondary antibody were purchased from Cell Signaling Technology, USA. Antibodies against Collagen I, PPAR*γ*, *β*-catenin, CD29, CD34, CD44, and CD45 were purchased from Abcam, UK. Reagent Sources including LY294002 (PI3K/Akt inhibitor), LiCl, dexamethasone, 3-isobutyl-1-methylxanthine (IBMX), indomethacin, and insulin were purchased from Sigma, USA. The enhanced chemiluminescence (ECL) detection substrate was purchased from Thermo Fisher Scientific, USA.

### 2.2. Isolation, Expansion, and Identification of BMSCs

Animal study was approved by the animal research committee of PLA Second Military Medical University. Neonatal Sprague-Dawley rats (7 days) were used for the isolation of BMSCs. Bone marrow was isolated aseptically by flushing the femurs and tibias of rats and then suspended in DMEM/F12 supplemented with 10% fetal bovine serum (FBS) and 1% penicillin-streptomycin in a humidified atmosphere incubator containing 95% air and 5% CO_2_ at 37°C. After 4 days, nonadherent cells were removed and medium was changed. Cells were passaged as the first passage when they were 90–95% confluent by trypsinization and seeded at a density of approximately 10^4^ cells/cm^2^ on round glasses (*φ* = 25 mm, Thickness = 0.17 mm) covered with polylysine. BMSCs phenotypes were confirmed by flow cytometry and analysis of cell surface molecules. Briefly, cells at passage one were washed in PBS and incubated with PE-labeled anti-CD29 (1 : 5), anti-CD34 (1 : 100) anti-CD44 (1 : 50), and FITC-labeled anti-CD45 (1 : 50) for 30 minutes. Then the cells were analyzed on a Flow Cytometer (BD Biosciences). Apoptotic cells were excluded from analysis using propidium iodide (PI).

### 2.3. Groups and Parameter Settings

According to absence or presence of different levels of stress intervention and different medium cells cultured in, the cells were divided into experimental and control groups, respectively. Stress parameters were set as follows: 0.15 Hz × 8 cm as small-magnitude and 0.6 Hz × 8 cm as large-magnitude for 30 min. The general medium was DMEM/F12 supplemented with 10% FBS and 1% penicillin-streptomycin, while the adipogenic medium consisted of basic high glucose DMEM supplemented with 1.0 *μ*M dexamethasone, 0.5 mM IBMX, 200 *μ*M indomethacin, 10 *μ*M insulin, 10% FBS, and 1% penicillin-streptomycin.

### 2.4. Cyclic Mechanical Stress Application

**Figure 1:**
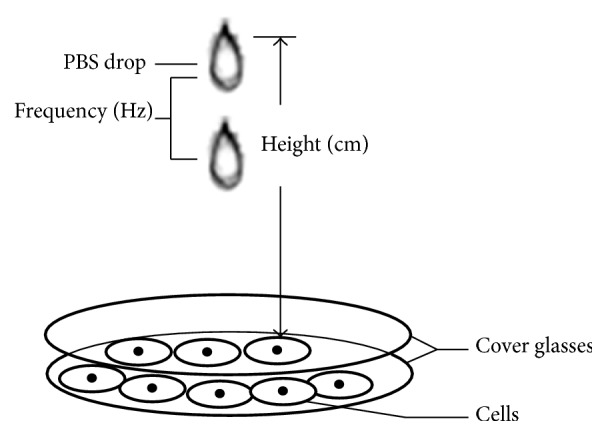
Diagrammatic representation for cyclic mechanical stress generated by PBS drops. Parameters of mechanical stress consisted of frequency (Hz) (controlled by drops per second) and height (cm) (perpendicular distance from drip nozzle of PBS to cover glass).

Cells on glasses of experimental groups were exposed to cyclic mechanical stress when they were 90–95% confluent. Briefly, thin cover glass plates were placed over the confluent cell layers on the glasses. The cyclic mechanical stress was adjusted by drops of phosphate buffer solution (PBS) to the glass plates. Cells were subjected to different frequency (Hz) (controlled by drops per second) and height (cm) of mechanical stress (Figure 1). Control cells were covered with a thin glass plate without any liquid dropping on it.

### 2.5. PI3K/Akt/GSK-3*β*/*β*-Catenin Signaling Modulation

For inhibition of PI3K/Akt signaling, cells were seeded on glasses as described and when they were 90–95% confluent, the medium was changed by DMEM/F12 with or without 10 *μ*M LY294002 for 1 h followed by mechanical stimulation (0.15 Hz × 8 cm) for 30 min. Cells without stress intervention were used as controls. Then cells were collected and protein expression levels of signaling pathway (p-Akt, Akt, GSK-3*β*, and *β*-catenin) were measured by Western blot. For activation of *β*-catenin, cells were cultured with either 10 mM LiCl or 10 mM sodium chloride (NaCl) (control) in adipogenic medium for 7 days. Then expression levels of adipogenic and osteogenic marker proteins in BMSCs were examined by Western blot.

### 2.6. Western Blot

Total proteins were extracted from cells by using lysis buffer (Beyotime, Shanghai, China), according to the manufacturer's instructions. Proteins from each sample were separated using 10% SDS-PAGE gels and transferred to polyvinylidene fluoride (PVDF) membranes using an electroblotting apparatus (Bio-Rad, Hercules, CA, USA). After blocking in 5% nonfat milk/TBS-Tween 20 (TBST) solution at room temperature for 1 h, membranes were then incubated with primary antibodies specific for Runx2 (1 : 1000), PPAR*γ* (1 : 1000), Collagen I (1 : 1000), C/EBP*α* (1 : 1000), p-Akt (1 : 1000), Akt (1 : 1000), GSK-3*β* (1 : 1000), *β*-catenin (1 : 1000), and *β*-actin(1 : 2000) at 4°C overnight. After washing with TBST, blots were then incubated at room temperature for 1 h with HRP-conjugated secondary antibodies diluted to 1 : 5000 in 5% nonfat milk/TBST. Protein bands were visualized using ECL reagents according to the manufacturer's instructions. The intensity values of each phosphorylated kinase were normalized to the corresponding total protein bands. Unless otherwise stated, *β*-actin was used as an internal control.

### 2.7. Histological Staining

Accumulation of lipid droplets was used to denote adipogenic differentiation of BMSCs. After fixation in 4% paraformaldehyde, cells were rinsed with 60% isopropanol, followed by freshly prepared Oil Red O solution (Sigma, 0.3% in isopropanol mixed 3 : 2 with deionized water).

### 2.8. Statistical Analysis

All data were presented as means ± standard deviation (SD). Statistical significance was determined using single-factor analysis of variance (ANOVA) followed by post hoc Bonferroni test to compare between groups. *P* < 0.05 was considered statistically significant.

## 3. Results

### 3.1. BMSCs Identification

**Figure 2:**
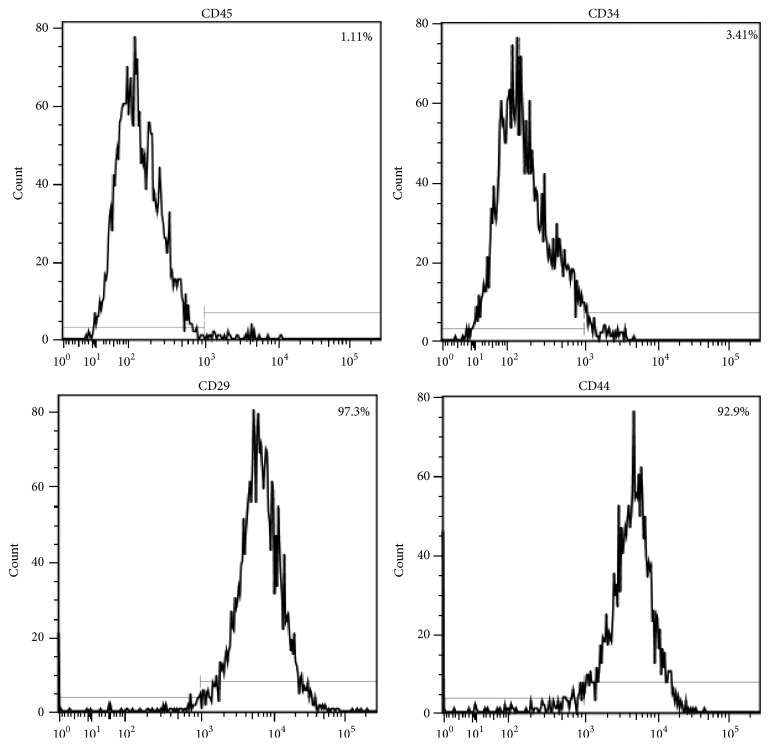
Flow cytometric analysis of surface molecule expression in BMSCs. Cells were positive for the surface antigens CD29 (97.3%) and CD44 (92.9%) and negative for CD34 (3.41%) and CD45 (1.11%).

BMSCs identification was done by the phenotypic analysis of the cells. Flow cytometric characterization analysis showed that the cells were homogenously positive for CD29 (97.3%) and CD44 (92.9%) and negative for CD34 (3.41%) and CD45 (1.11%) (Figure 2).

### 3.2. Effects of Mechanical Stress on BMSCs Differentiation

**Figure 3:**
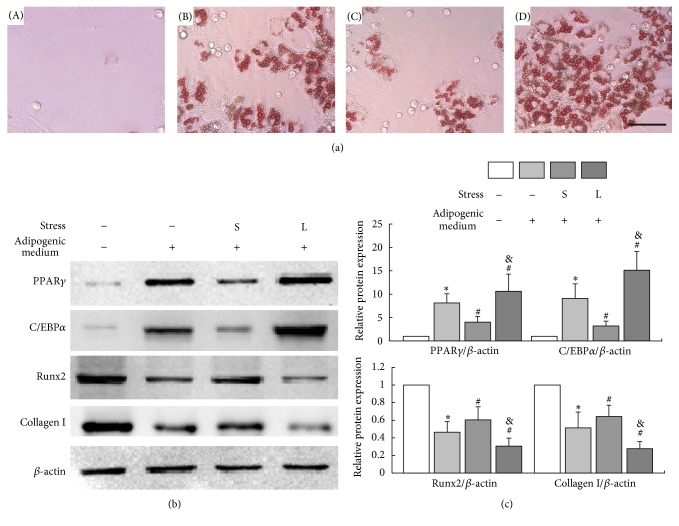
Effects of different levels of mechanical stress on BMSCs differentiation. BMSCs were exposed to different levels of mechanical stress for 30 min and cultured in adipogenic medium or general medium for 7 days. (a) The adipogenesis of BMSCs was confirmed by Oil Red O staining. (A) No obvious intracellular lipid droplet formed in the control group; (B) a moderate amount of intracellular lipid droplet formed in the group of unloading + adipogenic medium; (C) a slight amount of intracellular lipid droplet formed in the small-magnitude stress + adipogenic medium group; (D) a substantial amount of intracellular lipid droplet formed in the large-magnitude stress + adipogenic medium group. Bars = 50 *μ*m. (b) The expression levels of Runx2, Collagen I, C/EBP*α*, and PPAR*γ* in BMSCs were examined by Western blot. (c) Quantitative analysis. ^*∗*^*P* < 0.05 versus control group; ^#^*P* < 0.05 versus unloading + adipogenic medium group; ^&^*P* < 0.05 versus small-magnitude stress + adipogenic medium group. Each value was presented as mean ± SD of three separate experiments and data of the treatment group was expressed as fold change versus that of control group (labeled as “1.00”). S represented small-magnitude stress; L represented large-magnitude stress. Cells cultured in general medium without mechanical loading as control.

The adipogenesis of BMSCs was confirmed by Oil Red O staining of the droplets in cells. Significant development of lipid droplets was observed in BMSCs after exposure to adipogenic medium for 7 days compared with control groups. Application of small-magnitude mechanical stress reduced intracellular lipid droplets while large-magnitude mechanical stress led to an increasing of lipid droplets compared with unstressed groups (Figure 3(a)).

The expressions of Runx2, Collagen I, C/EBP*α*, and PPAR*γ* in BMSCs that were cultured for 7 days with or without mechanical stress were determined by Western blot. The results showed that compared with that of control, Runx2 and Collagen I were upregulated (*P* < 0.05) while C/EBP*α* and PPAR*γ* were downregulated, respectively (*P* < 0.05) after BMSCs were cultured in adipogenic medium for 7 days (Figures 3(b) and 3(c)).

After culture in adipogenic medium for 7 days, small-magnitude stress stimulation led to a significant downregulation of C/EBP*α* and PPAR*γ* and upregulation of Runx2 and Collagen I, respectively (*P* < 0.05) in BMSCs compared with unstressed groups. However, as the mechanical stress increased, the effect was reversed. Expressions of C/EBP*α* and PPAR*γ* in BMSCs increased significantly (*P* < 0.05) which were even higher than that of unstressed groups. Additionally, large-magnitude stress resulted in decreased expressions of Runx2 and Collagen I when compared with small-magnitude stress groups and unstressed groups, respectively (*P* < 0.05) (Figures 3(b) and 3(c)).

### 3.3. Effect of Mechanical Stress on Activation of PI3K/Akt/GSK-3*β*/*β*-Catenin Signaling

**Figure 4:**
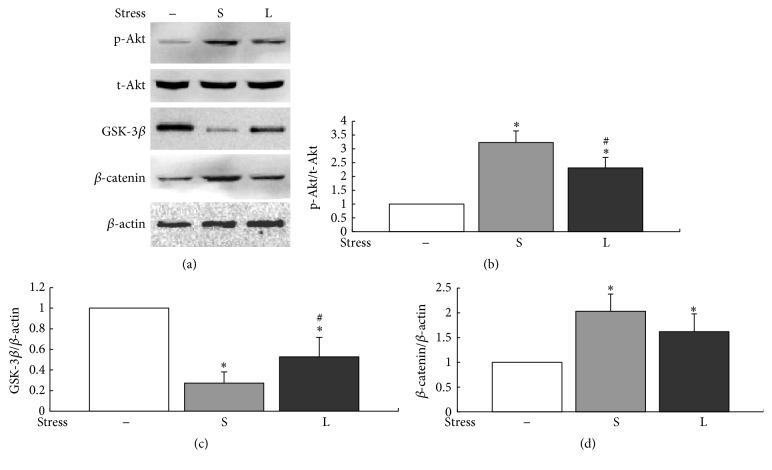
Effects of mechanical stress on PI3K/Akt/GSK-3*β*/*β*-catenin signaling pathway. BMSCs were stimulated with different levels of mechanical stress for 30 min. (a) Expression levels of PI3K/Akt/GSK-3*β*/*β*-catenin signaling proteins were detected by Western blot. (b–d) Quantitative analysis. ^*∗*^*P* < 0.05 versus control group; ^#^*P* < 0.05 versus small-magnitude stress group. Each value was presented as mean ± SD of three separate experiments and data of the treatment group was expressed as fold change versus that of control group (labeled as “1.00”). S represented small-magnitude stress; L represented large-magnitude stress.

The activation of PI3K/Akt/GSK-3*β*/*β*-catenin signaling was evaluated by Western blot analysis. The results showed that small-magnitude stress significantly upregulated the levels of p-Akt and *β*-catenin and inhibited the expression of GSK-3*β* compared with control cells (*P* < 0.05). In contrast, with the increasing of mechanical forces, levels of p-Akt (*P* < 0.05) and *β*-catenin decreased and GSK-3*β* (*P* < 0.05) increased, respectively (Figure 4). These results reflected that the manner of PI3K/Akt/GSK-3*β*/*β*-catenin signaling activation in BMSCs depended on the levels of mechanical stress loaded on them.

### 3.4. *β*-Catenin Induces Osteogenesis and Inhibits Adipogenesis of BMSCs

**Figure 5:**
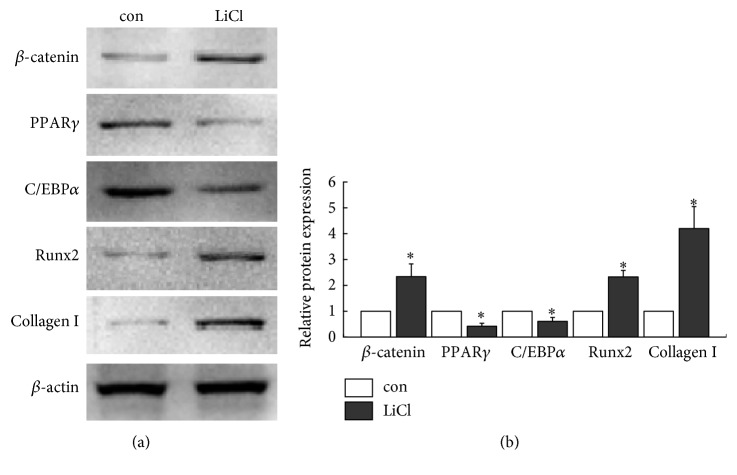
Effects of activation of *β*-catenin on BMSCs differentiation. (a) Cells were incubated with or without 10 mM LiCl for 24 h. *β*-Catenin in BMSCs was detected by Western blot. Cells were cultured in adipogenic medium for 7 days with or without LiCl. Expression levels of Runx2, Collagen I, C/EBP*α*, and PPAR*γ* were detected by Western blot. (b) Quantitative analysis. ^*∗*^*P* < 0.05 versus control group. Each value was presented as mean ± SD of three separate experiments and data of the treatment group was expressed as fold change versus that of control group (labeled as “1.00”).

Firstly, activation of *β*-catenin in BMSCs was investigated after treatment with LiCl. Western blot revealed that levels of *β*-catenin significantly increased after cells were treated with LiCl for 24 h (*P* < 0.05), suggesting that *β*-catenin could be activated by LiCl. To explore the influence of *β*-catenin on the differentiation of BMSCs, cells were cultured in adipogenic medium for a 7-day period in presence or absence of LiCl and adipogenic and osteogenic marker proteins in BMSCs were analyzed by Western blot. As shown in Figure 5, the results demonstrated that expressions of Runx2 and Collagen I increased with LiCl treatment (*P* < 0.05), accompanied by reduction of C/EBP*α* and PPAR*γ* expressions in BMSCs (*P* < 0.05). In all experiments, NaCl acted as a negative control for LiCl, which had no effect on *β*-catenin levels or differentiation of BMSCs [24].

### 3.5. *β*-Catenin-Regulated Different Differentiation in BMSCs Is PI3K/Akt-Dependent

**Figure 6:**
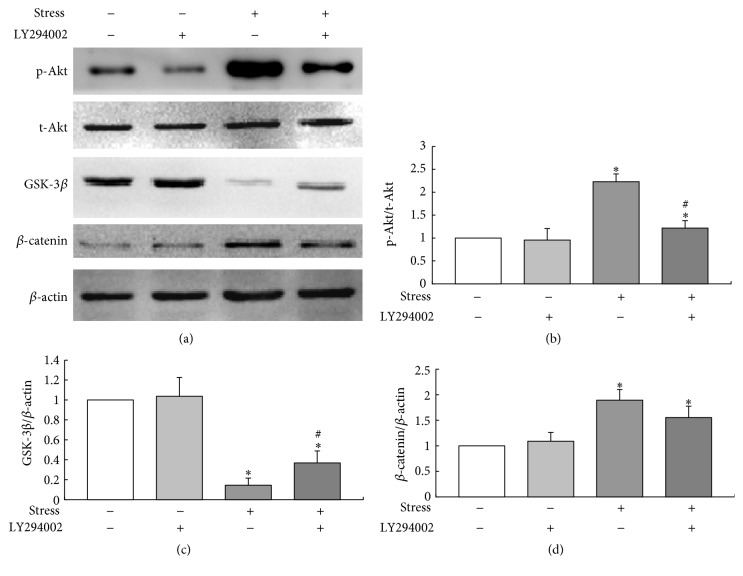
Activation of PI3K/Akt/GSK-3*β*/*β*-catenin signaling by the treatment with LY294002 and mechanical stress. MSCs were exposed to mechanical stress (0.15 Hz × 8 cm) for 30 min in the presence or absence of LY294002. (a) Western blot analysis of the protein levels of p-Akt, GSK-3*β*, and *β*-catenin. (b–d) Quantitative analysis. ^*∗*^*P* < 0.05 versus control group; ^#^*P* < 0.05 versus stress without inhibitor group. Each value was presented as mean ± SD of three separate experiments and data of the treatment group was expressed as fold change versus that of control group (labeled as “1.00”).

Pretreatment with 10 *μ*M LY294002 significantly blocked the mechanical stress-induced phosphorylation of Akt compared to the stress loaded cells without inhibitor (*P* < 0.05). Furthermore, inhibition of PI3K/Akt signaling partially blocked the expression of *β*-catenin by inactivation of GSK-3*β* (*P* < 0.05) under mechanical stress. However, LY294002 itself did not necessarily lead to any significant changes of p-Akt, *β*-catenin, and GSK-3*β* in the absence of mechanical stimulation (Figure 6). Taken together, the above findings indicated that mechanical stress regulated *β*-catenin via a PI3K/Akt-dependent signaling pathway.

## 4. Discussion

Although factors such as magnitude, frequency, and application time varied between studies, different types of mechanical stimuli, including tension [25–27], compression [8, 28], fluid shear stress [6], and hydrostatic pressure [29] have been confirmed to induce osteogenic differentiation with or without inhibition of adipogenesis of MSCs. However, MSCs experiencing tension at high levels exhibit reduced expression of osteogenic markers such as Runx2, alkaline phosphatase (ALP), and Collagen I [30]. In previous studies, mechanical loading was reported to inhibit the adipogenic commitment of MSCs. Findings of Yanagisawa et al. demonstrated that a high compressive force converts the differentiation pathway of C2C12 cells into that of the adipogenic lineage by upregulating the expression of PPAR*γ* [28]. However, this interesting phenomenon was just reported without discussion of any mechanism and to date little information is available regarding the effect of mechanical stress on the enhanced adipogenesis of MSCs. The results of the present study confirmed the hypothesis that the osteogenic and adipogenic features of BMSCs changed as the levels of mechanical stress increased and the effect was partially mediated by the PI3K/Akt/GSK-3*β*/*β*-catenin signaling pathway.

Adipogenesis is driven by a complex signaling pathway consisting of several key transcription factors including PPAR*γ* and C/EBP*α* [11]. PPAR*γ* is regarded as the central regulator of adipogenesis because no other factors have yet been reported to induce adipogenesis without it [31]. Several critical transcription factors such as Runx2, Osterix, and DLX2 play important roles in osteogenesis [23]. Of these, Runx2 is essential for the commitment of MSCs to the osteoblast lineage as Runx2 controls other osteoblast-related genes such as Osterix and Collagen I [32] as well as autoregulating itself [33]. Therefore, we investigated the commitment of MSCs to the osteoblast and adipocyte lineages by detecting expressions of PPAR*γ*, C/EBP*α*, Runx2, and Collagen I in the present study. In monolayer culture, BMSCs tend to differentiate into osteoblasts spontaneously instead of adipocytes when cultured in general medium. In order to investigate the effect of mechanical loading on the adipogenic differentiation of BMSCs, cells were cultured in adipogenic medium after mechanical application. By examining the specific markers, we demonstrated that large-magnitude mechanical loading increased adipogenesis and inhibited osteogenesis of MSCs, which was in accord with the findings of Yanagisawa et al. [28].

Cytoskeletal and focal adhesion have been reported to play an important role in mechanotransduction and to contribute to MSCs differentiation [1]. Cellular tension is generated through the interface of the external cellular environment with the internal actin cytoskeleton via integrins [34, 35]. Stimulation such as mechanical stress activates integrins, which results in phosphorylation of focal adhesion kinase and activation of a series of signaling pathways including PI3K/Akt, mitogen-activated protein kinases (MAPKs), protein kinase C, and GTPases of the Rho family [36]. Of the several signaling pathways, the PI3K/Akt pathway attracted our attention since we have previously examined the response of this pathway to different levels of cyclic mechanical stress and demonstrated that Akt is activated by small-magnitude mechanical stress, while phosphorylation of Akt is significantly reduced when large-magnitude mechanical stress is applied [20]. In the present report, we chose to subject the cells to two representative levels of mechanical stress, 0.15 Hz × 8 cm as small-magnitude and 0.6 Hz × 8 cm as large-magnitude. Our results showed the same trend of phosphorylation of Akt as our previous study.

The differentiation of MSCs is an intensely complex process, involving a large number of signaling pathways, cytokines, and transcription factors. Studies in recent years have demonstrated that a number of critical signaling pathways mediate MSCs differentiation, including transforming growth factor-beta (TGF-*β*)/bone morphogenic protein (BMP), MAPKs, wingless-type MMTV integration site (Wnt) signaling, Hedgehogs (Hh), Notch, and RhoA/ROCK pathways [22, 37, 38]. Wnt signaling is an important pathway that regulates osteogenesis. In the canonical Wnt pathway, *β*-catenin acts as a key transcriptional coactivator and transmits extracellular signals to the nucleus to activate target genes [39]. When the Wnt signaling pathway is inactivated, GSK-3*β* is active and *β*-catenin is constitutively phosphorylated by a destruction complex composed of Axin, APC, and GSK-3*β*, resulting in low cellular levels of *β*-catenin. Inactivation of GSK-3*β* induces the accumulation of cytosolic *β*-catenin and translocation to the nucleus to activate downstream target genes such as Runx2 and PPAR*γ* [39–41]. In MSCs, increased osteogenesis and repression of adipogenesis are dependent on activation of *β*-catenin by inhibition of GSK-3*β*. Either GSK-3*β* inactivation or *β*-catenin activation block the ability of mechanical signals to induce adipogenesis [42]. It has been reported that inactivation of *β*-catenin in MSCs results in decreased osteogenic differentiation in vivo and in vitro [43]. Results of a study by Sen et al. showed that inhibition of GSK-3*β* is essential for mechanical inhibition of adipogenesis [44]. Our results revealed that upregulation of *β*-catenin enhanced osteogenesis, while inhibiting adipogenesis of MSCs, even under adipogenic culture conditions, which is in accordance with their studies.

The PI3K/Akt pathway is reported to be activated by multiple types of mechanical stress [16, 45], which can inhibit GSK-3*β* and activate *β*-catenin [46]. Our present study showed that the changes of GSK-3*β* and *β*-catenin under different levels of mechanical loading were in response to changes in Akt. Akt activation was responsible for the consequent GSK-3*β* inhibition, as the expression of GSK-3*β* increased significantly in the presence of PI3K/Akt inhibitor.

Taken together, these results suggest that osteogenic and adipogenic differentiation of BMSCs induced by mechanical stress is regulated by the PI3KAkt/GSK-3*β*/*β*-catenin signaling pathway. However, we hypothesized that the effect of changes from the osteogenic to the adipogenic differentiation of BMSCs when mechanical stress increased is partially mediated by the pathway. In fact, decreased activation of *β*-catenin by large-magnitude mechanical stress could indeed lead to increased adipogenic differentiation relative to osteogenesis, but the phenomenon that large-magnitude mechanical stress induced more adipogenesis and less osteogenesis than that in the control group indicated that there were some other signaling pathways participating in the process. Specifically, mechanical loading leads to activation of the MAPKs, resulting in increased Runx2 and subsequent expression of osteoblast-specific genes [47, 48]. Furthermore, it is reported that several pathways mediate osteogenic differentiation of MSCs, including activation of ERK, PI3K/Akt [44], and insulin-like growth factor 1 (IGF1) signaling [49]. Our future studies will focus on other signaling pathways such as MAPKs and TGF-*β*/BMP signaling and related downstream key factors that may mediate the regulation of stress-induced differentiation of MSCs, which will promote our understanding of the networks of signaling pathways involved in this process and thus make beneficial use of appropriate mechanical stress and avoid the deleterious effects of pathological forces.
